# Association of Toll-like receptors polymorphisms with the risk of acute lymphoblastic leukemia in the Brazilian Amazon

**DOI:** 10.1038/s41598-022-19130-7

**Published:** 2022-09-07

**Authors:** Lilyane Amorim Xabregas, Fabíola Silva Alves Hanna, Fábio Magalhães-Gama, Gláucia Lima Souza, Daniele Sá Pereira, Amanda Barros de Lima, Diana Mota Toro, Mirian Rodrigues Ribeiro Santiago, Leny Nascimento da Motta Passos, Andréa Monteiro Tarragô, Adriana Malheiro, Allyson Guimarães Costa

**Affiliations:** 1 Programa de Pós-Graduação em Ciências Aplicadas à Hematologia, Universidade do Estado do Amazonas (UEA), Manaus, AM Brazil; 2 Diretoria de Ensino e Pesquisa, Fundação Hospitalar de Hematologia e Hemoterapia do Amazonas (HEMOAM), Av. Constantino Nery, 4397, Chapada, Manaus, AM 69050-001 Brazil; 3 Programa de Pós-Graduação em Imunologia Básica e Aplicada, Instituto de Ciências Biológicas, Universidade Federal do Amazonas (UFAM), Manaus, AM Brazil; 4 Programa de Pós-Graduação em Ciências da Saúde, Instituto René Rachou – Fundação Oswaldo Cruz (FIOCRUZ) Minas, Belo Horizonte, Brazil; 5 Rede Genômica de Vigilância em Saúde do Amazonas (REGESAM), Manaus, AM Brazil; 6 Programa de Pós-Graduação em Medicina Tropical, Universidade do Estado do Amazonas (UEA), Manaus, AM Brazil; 7 Escola de Enfermagem de Manaus, Universidade Federal do Amazonas, Manaus, AM Brazil

**Keywords:** Immunology, Genetics, Immunogenetics

## Abstract

Acute lymphoblastic leukemia (ALL) is the most common hematologic malignancy in children in childhood. Single-nucleotide polymorphism (SNPs) in key molecules of the immune system, such as Toll-like receptors (TLRs) and CD14 molecules, are associated with the development of several diseases. However, their role in ALL is unknown. A case–control study was performed with 152 ALL patients and 187 healthy individuals to investigate the role of SNPs in TLRs and the CD14 gene in ALL. In this study, *TLR6 C* > *T rs5743810* [OR: 3.20, 95% CI: 1.11–9.17, *p* = 0.003) and *TLR9* C > T *rs187084* (OR: 2.29, 95% CI: 1.23–4.26, *p* = 0.000) seems to be a risk for development of ALL. In addition, the *TLR1 T* > *G rs5743618* and *TLR6 C* > *T rs5743810* polymorphisms with protection against death (OR: 0.17, 95% IC: 0.04–0.79, *p* = 0.008; OR: 0.48, 95% IC: 0.24–0.94, *p* = 0.031, respectively). Our results show that SNPs in *TLRs* genes may be involved in the pathogenesis of ALL and may influence clinical prognosis; however, further studies are necessary to elucidate the role of *TLR1, TLR4*, *TLR5, TLR6, TLR9* and *CD14* polymorphisms in this disease.

## Introduction

Leukemia (acute and chronic) represents the 10th most frequent cause of cancer worldwide^1^. In Brazil, for the triennium 2020–2022, approximately 10,810 new cases in women and men are expected and approximately 300 new cases in North region of Brazil, where leukemia (acute and chronic) is the fifth most frequent cancer^2,3^. The most common type of leukemia in childhood is acute lymphoblastic leukemia (ALL) with a prevalence up to 25% of cancers in children who are under the age of 15 years^4^.

The neoplastic process results from genetic errors that contribute to blocking cell maturation and accumulation of leukemic clones (blasts) in the bone marrow microenvironment. Its etiology is still unknown; however, some risk factors are associated, including environmental, genetic and infectious factors^5,6^. Evidence from previous studies suggests that ALL is related to a deficit in immune system regulation in early childhood^7–12^. Furthermore, it is suggested that polymorphisms or genetic variations in the genes of molecules that are important in the development and progression of diseases may be important factors in the increase of intrinsic biological differences, influencing clinically distinct results and conferring genetic susceptibility to cancer^13,14^.

Toll-like receptors make up the main family of pattern recognition receptors (PRRs) of the innate immune system, and are involved in fighting pathogens and inflammation, and recognizing pathogen-associated molecular patterns (PAMPs) and damage-associated molecular patterns (DAMPs), which thus modulates the immune response via the activation of cells that mediate the immune response. In addition, TLRs are vital molecules in regulating the activation of adaptive immunity and are essential in preventing and curing cancer^15–18^. CD14 normally acts as a receptor for lipopolysaccharide (LPS) and LPS-binding proteins, which are responsible for IL-1, IL-6, and TNF-α inflammatory cytokine production. Studies have reported that CD14 may be involved in development of cancers such as acute lymphoblastic leukemia^19^.

Single-nucleotide polymorphism (SNPs) in TLRs and CD14 genes have been identified in different types of tumors, both in solid tumors and hematologic malignancies^13,20–23^. However, the role of immunogenic genes that play a crucial role in ALL is still poorly understood. In this study, we demonstrated the role of *TLR1, TLR4*, *TLR5, TLR6, TLR9,* and CD14 co-receptor polymorphisms in patients diagnosed with ALL.

## Results

### General characteristics of the study population

As shown in Table 1, the male gender was predominant in both groups with the median age 30 years for the control group and 12 years for case study group, 133 (71%) and 95 (62%), respectively. Most of the ALL patients had B-ALL (84%) and lived in Manaus (87%). A total of 64 (42%) had infectious comorbidities on diagnosis, 121 (80%) relapsed after induction therapy and 70 (46%) died. These patients also showed a median hemoglobin concentration of 8.6 g/dL, hematocrit of 25.1 g/dL, WBC 9.435/mm^3^ and platelets 58.000/mm^3^.

**Table 1:** Epidemiological and clinical characteristics of the study population.

Variables	Control group	ALL cases
	(n = 187)	(n = 152)
Age, years (median [IQR])	30 [23–39]	12 [4–18]
**Gender**		
Male, n (%)	133 (71%)	94 (62%)
Female, n (%)	54 (29%)	58 (38%)
**Ethnicity**		
Non-hispanic white	39 (21%)	19 (12%)
Multiracial	130 (69%)	130 (86%)
Black	7 (4%)	–
Indigenous	9 (5%)	–
East Asian	2 (1%)	3 (2%)
**Comorbidities**	–	
Yes, n (%)	–	67 (44%)
Infectious diseases, n (%)	–	64 (42%)
Others, n (%)	–	3 (2%)
No, n (%)	–	85 (56%)
**Immunophenotype**		
B-ALL		127 (84%)
T-ALL	–	25 (16%)
**Residence**	–	
Manaus	187 (100%)	87 (57%)
Interior of Amazonas state	–	53 (35%)
Other state	–	12 (8%)
**Relapse**	–	
Yes, n (%)	–	121 (80%)
No, n (%)	–	31 (20%)
**Death**	–	
Yes, n (%)	–	70 (46%)
No, n (%)	–	82 (54%)
Hemoglobin, g/dL (median [IQR])	–	8.6 [6.5–10.1]
Hematocrit, % (median [IQR])	–	25.1 [20.2–30.7]
Leukocyte, unit × 10^3^/mm^3^ (median [IQR])	–	9435 [3.388–57.850]
Platelets, unit × 10^6^/mm^3^ (median [IQR])	–	58,000 [0–152, 800]

### *TLR6 C* > *T rs5743810* and *TLR9 C* > *T rs5743836* polymorphisms predict risk of acute lymphoblastic leukemia

In our analyses, only *TLR5 R* > *S rs5744105* (OR: 1.39, 95% CI: 1.03–1.89, *p* = 0.000) and *TLR6* C > T *rs5743810* (OR: 0.57, 95% CI: 0.34–0.95, *p* = 0.033) in case group deviated from Hardy–Weinberg equilibrium. In Table 2, it can be observed that the *TLR6 C* > *T rs5743810* polymorphism seems to be a risk for developing acute lymphoblastic leukemia [OR: 3.20, 95% CI: 1.11–9.17, *p* = 0.003 [Codominant model]), as well as *TLR9* C > T *rs187084* (OR: 2.29, 95% CI: 1.23–4.26, *p* = 0.000, OR_adj_: 2.07, 95% CI_adj_: 0.86–4.98, *p* = 0.008 [Codominant model]) after Bonferroni correction. The other SNPs and allele associations can be found in Supplementary Table 2. We performed a regression model by ancestrally but no difference was observed.

**Table 2:** Analysis of the association *TLR6 C* > *T rs5743810 and TLR9 C* > *T rs187084* with the risk of acute lymphoblastic leukemia.

Genetic models	Control group n = 187 (%)	ALL patients n = 152 (%)	OR^a^ (95% Cl)^b^	*p* value^c^	AIC^d^	OR (95% CI) adj^e^	*p* value adj	AIC
***TLR6 C* > *T rs5743810***								
Codominant								
TT	121 (65%)	107 (70%)						
CT	61 (32%)	31 (21%)	0.58 (0.35–0.96)	***0.003***	459.2	0.61 (0.32–1.17)	0.056	284.8
CC	5 (3%)	14 (9%)	3.20 (1.11–9.17)			3.35 (0.74–15.14)		
Dominant								
TT	121 (65%)	107 (70%)	0.78 (0.49–1.23)	0.284	467.6	0.77 (0.42–1.42)	0.408	287.9
CT-CC	66 (35%)	45 (30%)						
Recessive								
TT-CT	182 (97%)	138 (91%)						
CC	5 (3%)	14 (9%)	3.72 (1.31–10.58)	***0.008***	461.8	3.90 (0.87–17.48)	0.060	285.0
Overdominant								
TT-CC	126 (67%)	121 (80%)	0.53 (0.32–0.88)	***0.012***	462.5	0.56 (0.29–1.08)	0.080	285.5
CT	61 (33%)	31 (20%)						
Log-additive 0,1,2	187 (55%)	152 (45%)	1.03 (0.72–1.48)	0.864	468.7	1.00 (0.61–1.64)	0.990	288.5
***TLR9 C* > *T rs187084***								
Codominant								
TT	61 (33%)	50 (33%)						
CT	102 (54%)	57 (37%)	0.68 (0.42–1.12)	***0.000***	455.5	0.58 (0.30–1.14)	***0.008***	281.1
CC	24 (13%)	45 (30%)	2.29 (1.23–4.26)			2.07 (0.86–4.98)		
Dominant								
TT	61 (33%)	50 (33%)	0.99 (0.63–1.56)	0.957	470.3	0.82 (0.44–1.54)	0.544	288.3
CT-CC	126 (67%)	102 (67%)						
Recessive								
TT-CT	163 (87%)	107 (70%)						
CC	24 (13%)	45 (30%)	2.86 (1.64–4.96)	***0.000***	455.8	2.85 (1.30–6.24)	***0.007***	281.6
Overdominant								
TT-CC	85 (45%)	95 (62%)		***0.001***	460.5		***0.008***	281.8
CT	102 (55%)	57 (38%)	0.50 (0.32–0.77)			0.45 (0.25–0.82)		
Log-additive 0,1,2	187 (55%)	152 (45%)	1.38 (1.02–1.87)	0.035	465.9	1.26 (0.82–1.91)	0.287	287.6

^a^OR: Odds Ratio; ^b^95% confidence interval; ^c^*p* value: < 0.05; ^d^AIC: Akaike information criterion value; ^e^Adj: Adjusted for sex and age.

### Association of *TLR1 T* > *G rs5743618 and TLR6 C* > *T rs5743810* polymorphisms with death in acute lymphoblastic leukemia

In Table 3, the *TLR1 T* > *G rs5743618* polymorphism was associated with protection from death (OR: 0.48, 95% IC: 0.24–0.94), *p* = 0.031 [Overdominant model], 95% IC_adj_: 0.42, OR_adj_: 0.21–0.87, *p* = 0.017 [Overdominant model]), as well as *TLR6 C* > *T rs5743810* (OR: 0.17, 95% IC: 0.04–0.79, *p* = 0.008 [Recessive model], OR_adj_: 0.14, 95% IC_adj_: 0.03–0.69, *p* = 0.004 [Recessive model] after Bonferroni correction. No association was found for infectious comorbidities (Supplementary Table 4). The allelic frequencies from all SNPs are shown in Supplementary Table 5.

**Table 3:** Analysis of the association of single nucleotide polymorphisms (SNPs) in study with the risk of death in acute lymphoblastic leukemia patients.

Genetic models	Death							
	No n = 82 (%)	Yes n = 70 (%)	OR^a^ (95% Cl)^b^	*p value*^c^	AIC^d^	OR (95% CI) adj^e^	*p* value adj	AIC
***TLR1 T* > *G rs5743618***								
Codominant								
TT	42 (51%)	44 (63%)						
TG	36 (44%)	19 (27%)	0.50 (0.25–1.01)	0.071	210.5	0.45 (0.21–0.93)	*0.046*	203.4
GG	4 (5%)	7 (10%)	1.67 (0.46–6.12)			1.60 (0.42–6.11)		
Dominant								
TT	42 (51%)	44 (63%)	0.62 (0.32–1.19)	0.148	211.7	0.56 (0.28–1.11)	0.093	204.7
TG-GG	40 (49%)	26 (37%)						
Recessive								
TT-TG	78 (95%)	63 (90%)						
GG	4 (5%)	7 (10%)	2.17 (0.61–7.73)	0.223	212.3	2.16 (0.58–8.00)	0.240	206.1
Overdominant								
TT-GG	46 (56%)	51 (73%)	0.48 (0.24–0.94)	*0.031*	209,1	0.42 (0.21–0.87)	***0.017***	201.8
TG	36 (44%)	19 (27%)						
Log-additive 0,1,2	82 (54%)	70 (46%)	0.85 (0.51–1.41)	0.523	213.4	0.80 (0.47–1.36)	0.407	206.8
***TLR6 C* > *T rs5743810***								
Codominant								
TT	55 (67%)	52 (74%)						
CT	15 (18%)	16 (23%)	1.13 (0.51–2.51)	*0.028*	208. 7	1.03 (0.44–2.41)	***0.017***	201.4
CC	12 (15%)	2 (3%)	0.18 (0.04–0.83)			0.14 (0.03–0.70)		
Dominant								
TT	55 (67%)	52 (74%)						
CT-CC	27 (33%)	18 (26%)	0.71 (0.35–1.43)	0.330	212.8	0.61 (0.29–1.29)	0.195	205.8
Recessive								
TT-CT	70 (85%)	68 (97%)						
CC	12 (15%)	2 (3%)	0.17 (0.04–0.79)	***0.008***	206.8	0.14 (0.03–0.69)	***0.004***	199.4
Overdominant								
TT-CC	67 (82%)	54 (77%)						
CT	15 (18%)	16 (23%)	1.32 (0.60–2.92)	0.486	213.3	1.23 (0.53–2.83)	0.629	207.3
Log-additive 0,1,2	82 (54%)	70 (46%)	0.62 (0.37–1.05)	0.068	210.5	0.56 (0.32–0.97)	***0.032***	203.0

^a^OR: Odds Ratio; ^b^95% confidence interval; ^c^p value: < 0.05; ^d^AIC: Akaike information criterion value; ^e^Adj: Adjusted for sex and age.

Significant values are in italics.

## Discussion

Genetic variations in the genes of molecules that are important in the immune response that is involved with the progression of diseases may prove to be important factors in the amplification of intrinsic biological differences, thus influencing clinically distinct results and conferring genetic susceptibility to cancer^13,14^. Single-variant polymorphisms in Toll-like receptors generally altered the TLRs ability to recognize pathogens in two forms: first, by increasing the response against pathogens that contribute to persistent inflammation and cancer and, second, by decreasing their response that contributes to infection susceptibility, as we see in chronic lymphoblastic leukemia (CLL)^13,18,24–26^.

The state of Amazonas, located in the Amazonian region of Brazil, is known for its tropical climate and a highly heterogeneous population that is exposed to several pathogens. In addition, it has different endemic areas for infectious diseases, which could contribute to the modulation of the immune response and consequently the triggering of physiological, genetic, and hematological changes^27^. The biological profile of ALL is still little known, and has different patterns according to the geographic and ethnic regions of the world^27–30^.

It is important to note that the population in study is exclusively composed by mixed population. Variations in Brazilian population have been clearly described in literature with a high degree of admixture from Americans, African and/ or European ancestors^31^. Besides, the Amazon Region population has a high degree of inter-ethnic admixture due to the intense miscegenation process and the strong indigenous influence. In this study, it was found that the allelic frequency of *TLR1 T* > *G rs5743618*, *CD14 C* > *T rs2569191*, *TLR4 A* > *G rs4986790*, *TLR4 C* > *T rs4986791* was similar to the American population, *TLR6 C* > *T rs5743810* and *TLR9 C* > *T rs187084* to European population, *TLR9 C* > *T rs5743836* to African population and *TLR5 R* > *S rs5744105* to East Asian population^32^. Previous studies demonstrated that children with admixed ancestry have a high risk of developing ALL because of the Native American ancestry^33^, which is highly concentrated in the north of the country, within the Amazon region^34^ and might be one of the reasons to explain the number of cases in the region. However, more genetic studies in this population are required to confirm this hypothesis.

To our knowledge, this is the first report to investigate the possible association between polymorphisms in *TLR* and *CD14* genes and the susceptibility to acute lymphoblastic leukemia in the Brazilian Amazon region. In our study, *TLR6 C* > *T rs5743810* and *TLR9 C* > *T r5743836* polymorphisms seem to be a risk for developing acute lymphoblastic leukemia. TLR-6 is a partner of TLR-2, which is responsible for PAMP recognition of invading pathogens and induces inflammation through myeloid differentiation primary response protein 88 (MyD88) and TRAF6 mediated activation of NF-κappa-B (NF-kB). In addition, in most studies, *TLR6 C* > *T rs5743810* is associated with infectious diseases like tuberculosis in the African population and the induction of resistance to asthma in children^35^. In CLL, high *TLR6* expression was observed in cells of the patients^36^, but it is still not clear what the role of this polymorphism is in leukemia.

TLR-9 is found in the endoplasmic reticulum membrane and functions via the MyD88-dependent pathway, which leads to NF-kB activation, cytokine secretion, and inflammatory response. A meta-analysis showed that *TLR9* polymorphisms are associated with increased risk for cancer and some hematological neoplasms, such as non-Hodgkin’s lymphoma and acute myeloid leukemia (AML)^37–39^. In AML, the [C] allele of *TLR9 C* > *T rs187084* and [T] *TLR9 C* > *T rs5743836* are associated with disease development, as is the TT genotype of the *TLR9 C* > *T rs5743836* polymorphism with relapse episodes^40^. Additionally, the *TLR9* gene is significantly expressed in chronic lymphocytic leukemia cells^36^.

Since the exposure of hematopoietic stem cells (HSCs) to TLR ligands influences the cycling, differentiation, and their functions, chronic TLR stimulation occurs, which leads to the impairment of normal HSC repopulating activity. Furthermore, high TLR expression and signaling are associated with myelodysplastic syndromes (MDS), which are a group of hematological neoplasms with a high risk of transformation to acute leukemias^13,41^. Therefore, these studies suggested that TLRs seem to be involved in the chronic neoplastic process, but further studies are necessary to confirm this.

A study involving the Turkish population showed no association between *TLR4 A* > *G rs4986790* and TLR4 C > T *rs4986791* and the risk for ALL^42^. Interestingly, In a cohort of AML patients, both these SNPs were independent risk factors for the development of sepsis and pneumonia^43^. In our study, we did not observed association between SNPs in TLRs with comorbidities infectious and, unfortunately, it was not possible to determine the association between the SNPs with cause of death because of lack information. Polymorphisms in TLR4 are responsible for decreased recognition of ligands (e.g., LPS). Since homozygotes and heterozygotes are hyporesponsive to LPS stimulation, this polymorphism may be associated with episodes of sepsis^44^. In addition, in B-chronic lymphoblastic leukemia (B-CLL), the reduced *TLR4* expression was associated with increased risk of disease progression and a poor outcome, infectious episodes, and evolution to autoimmune diseases, which suggests an impaired innate immunity^45^. In the literature, this SNP is associated with the risk of cervical^46^, rectal and neck cancer^47^.

It is important to note that our study has some limitations: (i) the sample size is a factor that influences the non-significance of the variants under study, however, studies with a larger sample size are required to increase the statistical power; (ii) the cytokines quantification and gene expression would allow us to better understand the influence of these variants in acute lymphoblastic leukemia patients but we understand that their absence not necessary weak our conclusions; (iii) our control group was composed by blood donors (> 18 years). Although we have included this topic as a limitation of the study, we understand that when using children as a control group, results can be obtained that do not reflect reality, since they could develop the disease after the study occurs; (iv) we did not have access to cause of the death in patients during chemotherapy (disease progression, infection, toxicity and other reason); (v) our clinical data collection was retrospective, so it is possible, for example, that not all patients with infections were identified, since we considered as infected only those who had requests for the described pathogens. Another point is that this was not always requested or the diagnosis was not available. Moreover, the patient could have an infection with some untested pathogen. This leads us to think that the number of patients with infectious comorbidities may be higher.

## Material and methods

### Patients and controls

A total of 152 pediatric ALL patients diagnosed according to the criteria of the World Health Organization (WHO)^48^, who received treatment at Fundação Hospitalar de Hematologia e Hemoterapia do Amazonas (HEMOAM), 0 to 18 years old, either gender and unrelated were included in the case study group. In the control group, 187 blood donor candidates of either gender and age 18 to 65 years old and who were considered healthy according to the Brazilian Ministry of Health technical standards were included^49^.

### Genomic DNA extraction

Genomic DNA was extracted from 4 mL of peripheral whole blood at diagnosis using the illustra triplePrep Genomicprep DNA Extraction kits® (GE Healthcare Life Sciences) and BIOPUR Mini Spin plus® extraction kit (Mobius Life Science) for the case group and the QIAmp DNA kit (QIAGEN, Chatsworth, CA, USA) for the control group, following standard laboratory protocols.

### SNP selection and genotyping

Eight SNPs [*TLR1 I602S* (*rs5743618*), *TLR4 A299G* (*rs4986790*), *TLR4 T399I* (*rs4986791*), *TLR5 R392StopCodon* (*rs5744105*), *TLR6 S249P* (*rs5743810*), *TLR9-1237C/T* (*rs5743836*), *TLR9-1486 C/T* (*rs187084)* and *CD14-159* (*rs2569191*)] were selected according to previously reported hematological disease associations (Non-Hodgkin’s lymphoma and AML) and frequency in the Brazilian Amazon population^37,50–52^. Genotyping was performed using the polymerase chain reaction restriction fragment length polymorphism (PCR–RFLP) technique according to the protocol described by COSTA et al. (2017)^52^. The PCR reaction consisted of 2 μL of genomic DNA (20 ng) and 23 μL of amplification mixture containing 0.1 μL of Platinum Taq DNA Polymerase (2 UI), 2.5 μL of 10 × buffer (containing 100 mmol/L Tris–HCl [pH 8.3] and 500 mmol/L KCL), 2 μL of MgCl_2_ (1.5 mmol/L), 1 μL of dNTPs (40 mmol/L), 0.25 μL for each forward and reverse primer (0.25 pmol/L) and 16.9 μL of ultrapure water. A total of 10 μL of PCR product was digested with 5 U of the respective restriction endonuclease (New England Biolabs) and 10 × enzyme buffer according to the manufacturer's instructions. Primers, PCR cycling conditions, and restriction endonucleases are described in Supplementary Table 1. PCR–RFLP generated fragments were separated using 3–4% agarose gel electrophoresis stained with GelRed™ nucleic acid gel stain (Biotium, Hayward, CA, USA) and visualized on a UV light gel + DocXR system transilluminator (Bio-Rad Corporation, Hercules, CA, USA) with a photographic documentation system.

### Data collection

The collection of clinical and demographic data was obtained at diagnosis via a search in physical records of the Medical and Statistical Care System (SAME) at the HEMOAM Foundation. In this study, we used the Updated Guidance on the Reporting of Race and Ethnicityin Medical and Science Journals^53^. Infectious comorbidities were considered infections that serologically tested as IgG + and IgM + (cytomegalovirus, toxoplasmosis, rubella, varicella and parasitic diseases, among others) according to ALVES et al. (2021)^54^, and pancreatitis, hemorrhage, and sepsis were considered as “Others”. As the relapse criterion, patients who relapsed after induction therapy (35th day of treatment from Grupo Brasileiro de Tratamento das Leucemias Infantis [GBTLI] 2009 protocol)^55^ were included and, for death, patients who died within 5 years were included.

### Descriptive and statistical analysis

Comparison between groups was performed using Fisher’s exact test using GraphPad Prism v.5.0, with a significance level of 5%. Allele analysis was performed using the website https://ihg.helmholtz-muenchen.de/ihg/snps.html. A logistic regression analysis that was adjusted for sex and age was performed to find associations between the genotype frequencies and ALL, infectious comorbidities, and death using the package “SNPassoc” version 1.9-2 (https://cran.rproject.org/web/packages/SNPassoc/index.html) for R software version 3.4.3 (www.r-project.org). The best genetic model was performed using the Akaike information criterion (AIC). Hardy–Weinberg equilibrium was evaluated for all SNPs. Bonferroni correction for multiple tests was also performed and the *p*-value adjusted was reported in bold italic characters. The results were shown as the odds ratio (OR) and 95% confidence intervals (95% CI) from multivariate logistic regression analyses.

### Ethics approval and consent to participate

All protocols and consent forms were approved by the Research Ethics Committee at the HEMOAM Foundation (CEP/HEMOAM process 3.335.123/2019). The informed consent form was obtained from all patients and parents/legal guardians for minors in study. This study was carried out following the guidelines of the Declaration of Helsinki and Resolution 466/12 of the Brazilian National Health Council for research involving human beings.

## Conclusion

To our knowledge, this is the first study to describe the frequency of SNPs in the *TLR1, TLR4*, *TLR5, TLR6, TLR9* and *CD14* genes in patients with ALL in the Brazilian Amazon region. Our study demonstrated that *TLR6 C* > *T rs5743810* and *TLR9* C > T *rs5743836* polymorphisms are associated with the risk of acute lymphoblastic leukemia and *TLR1 T* > *G rs5743618* and *TLR9 C* > *T rs5743810* is involved with death. Further studies are necessary to elucidate the role of the *TLR1, TLR4*, *TLR5, TLR6, TLR9,* and *CD14* polymorphisms in the pathogenesis of leukemia.

## Supplementary Information

Supplementary Information 1.

Supplementary Information 2.

Supplementary Information 3.

Supplementary Information 4.

Supplementary Information 5.

## Data Availability

All data generated or analysed during this study are included in this published article.
