# Supplementary material for: Association of Toll-like receptors polymorphisms with the risk of acute lymphoblastic leukemia in the Brazilian Amazon
Source: Sci Rep. 2022 Sep 7;12:15159. doi: 10.1038/s41598-022-19130-7 (PMC9452670; doi:10.1038/s41598-022-19130-7)
Supplement: Supplementary file 1 — Supplementary Information 1. [file 41598_2022_19130_MOESM1_ESM.docx]

**Supplementary Table 1**. Description of the sequences of the primers, cycles, restriction enzymes and fragments of the study polymorphisms.

| SNP^a^ ID | Gene | Chromosome | Variation | Allele | Primer Sequence (5´- 3´) | PCR protocol | Restriction Enzyme | Fragments |
| --- | --- | --- | --- | --- | --- | --- | --- | --- |
| *rs5743618* | *TLR1* | Chr.4: 38797027 | *l602S*  (missense) | T>G | Forward:  GGAAAGTTATAGAGGAACCCT  Reverse:  CTTCACCCAGAAAGAATCGTGCC | 1 cycle per 95°C for 5’,  40 cycles of 95°C for 30’’,  55°C for 30’’ and 72°C for 30’’,  1 cycle of 72°C for 7’ | *AluI* | T: 280  G: 151+129 |
| *rs4986790* | *TLR4* | Chr.9: 117713024 | *A299G*  (missense) | A>G | Forward:  GATTAGCATACTTAGACTACCTCCATG  Reverse:  GATCAACTTCTGAAAAAGCATTCCCAC | 1 cycle of 95^0^C for 5’,  40 cycles of 95^0^C for 30’’,  56^0^C for 30’’ and 72^0^C for 30’’  1 cycle of 72^0^C for 7’ | *NcoI* | A: 259  G: 239+20 |
| *rs4986791* | *TLR4* | Chr.9: 117713324 | *T399l*  (missense) | C>T | Forward:  GGTTGCTGTTCTCAAAGTTTTGGGAGAA  Reverse:  ACCTGAAGACTGGAGAGTGAGTTAAATGCT | 1 cycle of 95^0^C for 5’,  40 cycles of 95^0^C for 15’’,  60^0^C for 15’’ and 72^0^C for 30’’  1 cycle of 72^0^C for 7’ | *Hinf-I* | C: 147  T: 96+51 |
| *rs5744105* | *TLR5* | Chr.1: 223142735 | *R392StopCodon*  (Íntron) | R>S | Forward:  GGTAGCCTACATTGATTTGC  Reverse:  GAGAATCTGGAGATGAGGTACCCG | 1 cycle of 95°C for 5’,  40 cycles of 95°C for 30’’,  62°C for 30’ and 72°C for 30’’,  1 cycle of 72°C for 7’ | *DdeI* | R: 277pb  S: 186+91 |
| *rs5743810* | *TLR6* | Chr.4: 38828729 | *S249P*  (missense) | C>T | Forward:  GCATTTCCAAGTCGTTTCTATGT  Reverse:  GCAAAAACCCTTCACCTTGTT | 1 cycle of 95°C for 5’.  40 cycles of 95°C for 30’’,  63°C for 30’’ and 72°C for 30’’,  1 cycle of 72°C for 7’ | *AvaII* | C: 210  T: 160+50 |
| *rs187084* | *TLR9* | Chr.3: 52227015 | *-1237*  (Íntron) | C>T | Forward:  CTGCTTGCAGTTGACTGTGT  Reverse:  ATGGGAGCAGAGACATAATGGA | 1 cycle of 95°C for 5’,  40 cycles of 95°C for 30’’,  59°C for 30’’ and 72°C for 45’’,  1 cycle of 72°C for 7’ | *BstNI* | C: 108+27  T: 60+48+27 |
| *rs5743836* | *TLR9* | Chr.3: 52226766 | *-1486*  (Íntron) | C>T | Forward:  TATCGTCTTATTCCCCTGCTGGAATGT  Reverse:  TGCCCAGAGCTGACTGCTGG | 1 cycle of 95°C for 5’,  40 cycles of 95°C for 30’’,  59°C for 30’’ and 72°C for 30’’, 72°C for 7’ | *AflII* | C: 145  T: 111+34 |
| *rs2569191* | *CD14* | Chr.5: 140634318 | *-159*  (intron) | C>T | Forward:  GTGCCAACAGATGAGGTTCAC  Reverse:  GCCTCTGACAGTTTATGTAATC | 1 cycle of 94ºC for 5’,  35 cycles of 94ºC for 30’’,  64ºC for 30’’ e 72ºC for 1’  1 cycle of 72ºC for 10’ | *Ava II* | C: 497  T: 353+144 |

^a^SNPs: Single nucleotide polymorphisms. ^b^rs: Reference Sequence.
