# Supplementary material for: Association of Toll-like receptors polymorphisms with the risk of acute lymphoblastic leukemia in the Brazilian Amazon
Source: Sci Rep. 2022 Sep 7;12:15159. doi: 10.1038/s41598-022-19130-7 (PMC9452670; doi:10.1038/s41598-022-19130-7)
Supplement: Supplementary file 2 — Supplementary Information 2. [file 41598_2022_19130_MOESM2_ESM.docx]

**Supplementary Table 2.** Analysis of the association of single nucleotide polymorphisms (SNPs) in study with acute lymphoblastic leukemia.

| Genetic  models | Controls  n=187 (%) | ALL cases  n=152 (%) | OR (95% Cl) | *p* value | AIC | OR (95% CI)  adj | *p* value  adj | AIC |
| --- | --- | --- | --- | --- | --- | --- | --- | --- |
|  | ***TLR1 T>G rs5743618*** | | | | | |  |  |
| Codominant |  |  |  |  |  |  |  |  |
| TT | 101 (54%) | 86 (57%) |  |  |  |  |  |  |
| TG | 74 (40%) | 55 (36%) | 0.87 (0.56 – 1.37) | 0.804 | 471.9 | 0.87 (0.47 – 1.61) | 0.640 | 289.8 |
| GG | 12 (6%) | 11 (7%) | 1.08 (0.45 – 2.56) |  |  | 1.56 (0.46 – 5.24) |  |  |
| Dominant |  |  |  |  |  |  |  |  |
| TT | 101 (54%) | 86 (57%) | 0.90 (0.59 – 1.39) | 0.636 | 470.1 | 0.95 (0.52 – 1.71) | 0.853 | 288.7 |
| TG-GG | 86 (46%) | 66 (43%) |  |  |  |  |  |  |
| Recessive |  |  |  |  |  |  |  |  |
| TT-TG | 175 (94%) | 141 (93%) |  |  |  |  |  |  |
| GG | 12 (6%) | 11 (7%) | 1.14 (0.49 – 2.66) | 0.765 | 470.2 | 1.66 (0.51– 5.41) | 0.403 | 288.0 |
| Overdominant |  |  |  |  |  |  |  |  |
| TT-GG | 113 (60%) | 97 (64%) | 0.87 (0.56 – 1.35) | 0.522 | 469.9 | 0.83 (0.45 – 1.51) | 0.540 | 288.3 |
| TG | 74 (40%) | 55 (36%) |  |  |  |  |  |  |
| Log-Additive  0,1,2 | 187 (55%) | 152 (45%) | 0.96 (0.68 – 1.35) | 0.796 | 470.3 | 1.05 (0.65 – 1.69) | 0.851 | 288.7 |
|  | ***CD14 C>T*** ***rs2569191*** | | | | | |  |  |
| Codominant |  |  |  |  |  |  |  |  |
| CC | 55 (30%) | 39 (26%) |  |  |  |  |  |  |
| CT | 93 (50%) | 85 (56%) | 1.29 (0.78 – 2.14) | 0.551 | 470.0 | 0.89 (0.45 – 1.77) | 0.813 | 289.6 |
| TT | 38 (20%) | 28 (18%) | 1.04 (0.55 – 1.97) |  |  | 0.76 (0.32 – 1.77) |  |  |
| Dominant |  |  |  |  |  |  |  |  |
| CC | 55 (30%) | 39 (26%) | 1.22 (0.75 –1.97) | 0.424 | 468.5 | 0.85 (0.44 – 1.63) | 0.623 | 288.7 |
| CT-TT | 131 (70%) | 113 (74%) |  |  |  |  |  |  |
| Recessive |  |  |  |  |  |  |  |  |
| CC-CT | 148 (80%) | 124 (82%) |  |  |  |  |  |  |
| TT | 38 (20%) | 28 (18%) | 0.88 (0.51– 1.51) | 0.642 | 468.9 | 0.82 (0.40 – 1.68) | 0.583 | 287.7 |
| Overdominant |  |  |  |  |  |  |  |  |
| CC-TT | 93 (50%) | 67 (44%) | 1.27 (0.83 – 1.95) | 0.278 | 468.0 | 1.00 (0.56 – 1.80) | 0.994 | 288.0 |
| CT | 93 (50%) | 85 (56%) |  |  |  |  |  |  |
| Log-Additive  0,1,2 | 186 (55%) | 152 (45%) | 1.04 (0.76 -1.43) | 0.799 | 469.1 | 0.87 (0.57 – 1.33) | 0.523 | 287.6 |
|  | ***TLR4 A>G rs4986790*** | | | | | |  |  |
| Codominant |  |  |  |  |  |  |  |  |
| AA | 177 (95%) | 144 (95%) |  |  |  |  |  |  |
| AG | 10 (5%) | 7 (4%) | 0.86 (0.32– 2.32) | 0.617 | 470.6 | 0.83 (0.19 – 3.57) | 0.925 | 290.6 |
| GG | 0 (0%) | 1 (1%) | - |  |  | - |  |  |
| Dominant |  |  |  |  |  |  |  |  |
| AA | 177 (95%) | 144 (95%) | 0.98 (0.38 – 2.56) | 0.972 | 470.3 | 0.85 (0.20 – 3.59) | 0.824 | 288.7 |
| AG-GG | 10 (5%) | 8 (5%) |  |  |  |  |  |  |
| Recessive |  |  |  |  |  |  |  |  |
| AA-AG | 187 (100%) | 151 (99%) |  |  |  |  |  |  |
| GG | 0 (0%) | 1 (1%) | - | - | - | - | - | - |
| Overdominant |  |  |  |  |  |  |  |  |
| AA-GG | 177 (95%) | 145 (95%) | 0.85 (0.32 – 2.30) | 0.755 | 470.2 | 0.83 (0.19 – 3.57) | 0.802 | 288.7 |
| AG | 10 (5%) | 7 (5%) |  |  |  |  |  |  |
| log-Addtive  0,1,2 | 187 (55%) | 152 (45%) | 1.10 (0.46 –2.65) | 0.617 | 470.3 | 0.87(0.22 – 3.52) | 0.849 | 288.7 |
|  | ***TLR4 C>T rs4986791*** | | | | | |  |  |
| Codominant |  |  | |  |  |  |  |  |
| CC | 180 (96%) | 143 (94%) |  |  |  |  |  |  |
| CT | 7 (4%) | 8 (5%) | 1.44 (0.51 – 4.06) | 0.429 | 470.3 | 1.61 (0.38 – 6.83) | 0.782 | 290.2 |
| TT | .0 (0%) | 1 (1%) | - |  |  | - |  |  |
| Dominant |  |  |  |  |  |  |  |  |
| CC | 180 (96%) | 143 (94%) | 1.62 (0.59 –4.45) | 0.349 | 469.5 | 1.63 (0.39 – 6.87) | 0.504 | 288.3 |
| CT-TT | 7 (4%) | 9 (6%) |  |  |  |  |  |  |
| Recessive |  |  |  |  |  |  |  |  |
| CC-CT | 187 (100%) | 151 (99%) |  |  |  |  |  |  |
| TT | 0 (0%) | 1 (1%) | - | - | - | - | - | - |
| Overdominant |  |  |  |  |  |  |  |  |
| CC-TT | 187 (100%) | 144 (95%) | 1.43 (0.51 – 4.03) | 0.500 | 469.9 | 1.61 (0.38 – 6.83) | 0.517 | 288.3 |
| CT | 0 (0%) | 8 (5%) |  |  |  |  |  |  |
| log-Additive  0,1,2 | 187 (55%) | 152 (45%) | 1.70 (0.66 -4.37) | 0.429 | 469.1 | 1.63 (0.39 – 6.80) | 0.496 | 288.3 |
|  | ***TLR5 R>S*** ***rs5744105*** | | | | | |  |  |
| Codominant |  |  |  |  |  |  |  |  |
| RR | 177 (95%) | 142 (93%) |  |  |  |  |  |  |
| RS | 10 (5%) | 10 (7%) | 1.25 (0.5– 3.08) | 0.633 | 470.1 | 1.47 (0.47 – 4.55) | 0.507 | 288.3 |
| log-Additive  0,1,2 | 187 (55%) | 152 (45%) | 1.25 (0.5 – 3.08) |  | 470.1 | 1.47(0.47 – 4.55) |  | 288.3 |
|  | ***TLR9 C>T rs187084*** | | | | | |  |  |
| Codominant |  |  |  |  |  |  |  |  |
| TT | 121(65%) | 1 (1%) |  |  |  |  |  |  |
| CT | 66 (35%) | 39 (25%) | 71.50 (9.60 – 532.28) | 3.122 | 156.1 | 81.03 (9.41– 697.97) | 1.540 | 93.6 |
| CC | 0 (0%) | 112 (74%) | - |  |  | - |  |  |
| Dominant |  |  |  |  |  |  |  |  |
| TT | 121 (65%) | 1 (1%) |  |  |  |  |  |  |
| CT-CC | 66 (35%) | 151 (99%) | 276.83 (37.88) | 8.214 | 282.2 | 235.01 (30.25) | 7.552 | 173.6 |
| Recessive |  |  |  |  |  |  |  |  |
| TT-CT | 187 (100%) | 40 (26%) |  |  |  |  |  |  |
| CC | 0 (0%) | 112 (74%) | - | - | - | - | - | - |
| Overdominant |  |  |  |  |  |  |  |  |
| TT-CC | 121 (65%) | 113 (74%) | 0.63 (0.39 – 1.01) | 5.523 | 466.7 | 0.93 (0.49 – 1.77) | 8.215 | 288.7 |
| CT | 66 (35%) | 39 (26%) |  |  |  |  |  |  |
| log-Additive  0,1,2 | 187 (55%) | 152 (45%) | 254.08 (35.40) | 3.122 | 156.6 | 407.05 (42.47) | 4.825 | 95.0 |

^a^Adjusted for sex and age; ^b^OR: Odds Ratio; ^c^p value: < 0.05; ^d^95% confidence interval; ^e^AIC: Akaike information criterion value.
