# Supplementary material for: Association of Toll-like receptors polymorphisms with the risk of acute lymphoblastic leukemia in the Brazilian Amazon
Source: Sci Rep. 2022 Sep 7;12:15159. doi: 10.1038/s41598-022-19130-7 (PMC9452670; doi:10.1038/s41598-022-19130-7)
Supplement: Supplementary file 3 — Supplementary Information 3. [file 41598_2022_19130_MOESM3_ESM.docx]

**Supplementary Table 3.** Analysis of the association of single nucleotide polymorphisms (SNPs) in study with the risk of death in acute lymphoblastic leukemia patients.

|  | Death | | | | | | | |
| --- | --- | --- | --- | --- | --- | --- | --- | --- |
| Genetic  models | **No**  **n= 82 (%)** | **Yes**  **n=70 (%)** | **OR (95% CI)** | ***p* value** | **AIC** | **OR (95% CI)**  **adj** | ***p* value**  **adj** | **AIC** |
| *CD14 C>T rs2569191* | | | | | | | | |
| Codominant |  |  |  |  |  |  |  |  |
| CC | 21 (26%) | 18 (26%) |  |  |  |  |  |  |
| CT | 46 (56%) | 39 (56%) | 0.99 (0.46 – 2.12) | 0.998 | 215.8 | 1.19 (0.53 – 2.65) | 0.912 | 209.3 |
| TT | 15 (18%) | 13 (18%) | 1.01 (0.38 – 2.68) |  |  | 1.10 (0.39 – 3.06) |  |  |
| Dominant |  |  |  |  |  |  |  |  |
| CC | 21 (26%) | 18 (26%) |  |  |  |  |  |  |
| CT-TT | 61 (74%) | 52 (74%) | 0.99 (0.48 – 2.06) | 0.988 | 213.8 | 1.17 (0.54 – 2.52) | 0.696 | 207.4 |
| Recessive |  |  |  |  |  |  |  |  |
| CC-TT | 67 (82%) | 57 (81%) |  |  |  |  |  |  |
| TT | 15 (18%) | 13 (19%) | 1.02 (0.45 – 2.32) | 0.964 | 213.8 | 0.97 (0.41 – 2.29) | 0.951 | 207.5 |
| Overdominant |  |  |  |  |  |  |  |  |
| CC-TT | 36 (44%) | 31 (44%) | 0.98 (0.52 – 1.87) | 0.962 | 213.8 | 1.14 (0.58 – 2.23) | 0.698 | 207.4 |
| CT | 46 (56%) | 39 (56%) |  |  |  |  |  |  |
| Log-Additive  0,1,2 | 82 (54%) | 70 (46%) | 1.00 (0.62 – 1.63) | 0.987 | 213.8 | 1.06 (0.64 – 1.76) | 0.824 | 207.5 |
| *TLR4 A>G rs4986790* | | | | | | | | |
| Codominant |  |  |  |  |  |  |  |  |
| AA | 78 (95%) | 66 (94%) |  |  |  |  |  |  |
| AG | 3 (4%) | 4 (6%) | 1.58 (0.34 – 7.29) | 0.840 | 214.2 | 1.90 (0.39 – 9.23) | 0.520 | 208.2 |
| GG | 1 (1%) | 0 (0%) | - | - | - | - | - | - |
| Dominant |  |  |  |  |  |  |  |  |
| AA | 78 (95%) | 66 (94%) | 1.18 (0.28 – 4.91) | 0.818 | 213.7 | 1.51 (0.35 – 6.57) | 0.585 | 207.2 |
| AG-GG | 4 (5%) | 4 (6%) |  |  |  |  |  |  |
| Recessive |  |  |  |  |  |  |  |  |
| AA-AG | 81 (99%) | 70 (100%) |  |  |  |  |  |  |
| GG | 1 (1%) | 0 (0%) | - | - | - | - | - | - |
| Overdominant |  |  |  |  |  |  |  |  |
| AA-GG | 79 (96%) | 66 (94%) | 1.60 (0.34 – 7.39) | 0.547 | 213.4 | 1.91 (0.39- 9.28) | 0.421 | 206.9 |
| AG | 3 (4%) | 4 (6%) |  |  |  |  |  |  |
| Log-Additive  0,1,2 | 82 (54%) | 70 (46%) | 0.95 (0.28 – 3.20) | 0.840 | 213.8 | 1.20 (0.34 – 4.23) | 0.773 | 207.4 |
| *TLR4 C>T rs4986791* | | | | | | | | |
| Codominant |  |  |  |  |  |  |  |  |
| CC | 75 (92%) | 68 (97%) |  |  |  |  |  |  |
| CT | 6 (7%) | 2 (3%) | 0.37 (0.07 – 1.88) | 0.288 | 212.9 | 0.36 (0.07 – 1.91) | 0.317 | 207.2 |
| TT | 1 (1%) | 0 (0%) | - |  |  | - |  |  |
| Dominant |  |  |  |  |  |  |  |  |
| CC | 75 (92%) | 68 (97%) | 0.32 (0.06 – 1.57) | 0.126 | 211.4 | 0.33 (0.06 – 1.71) | 0.154 | 205.5 |
| CT-TT | 7 (8%) | 2 (3%) |  |  |  |  |  |  |
| Recessive |  |  |  |  |  |  |  |  |
| CC-CT | 81 (99%) | 70 (100%) |  |  |  |  |  |  |
| TT | 1 (1%) | 0 (0%) | - | - | - | - | - | - |
| Overdominant |  |  |  |  |  |  |  |  |
| CC-TT | 76 (93%) | 68 (97%) | 0.37 (0.07 – 1.91) | 0.207 | 212.2 | 0.36 (0.07- 1.93) | 0.205 | 205.9 |
| CT | 6 (7%) | 2 (3%) |  |  |  |  |  |  |
| log-Addtive  0,1,2 | 82 (54%) | 70 (46%) | 0.33 (0.07 – 1.49) | 0.288 | 211.1 | 0.34(0.07 – 1.66) | 0.138 | 205.3 |
| *TLR5 R>S rs5744105* | | | | | | | | |
| Codominant |  |  |  |  |  |  |  |  |
| RR | 77 (94%) | 65 (93%) | 1.18 (0.33 – 4.27) | 0.795 | 213.7 | 0.93 (0.24 – 3.57) | 0.914 | 207.5 |
| RS | 5 (6%) | 5 (7%) |  |  |  |  |  |  |
| log-Additive  0,1,2 | 82 (54%) | 70 (46%) | 1.18 (0.33 – 4.27) | 0.795 | 213.7 | 0.93 (0.24 – 3.57) | 0.914 | 207.5 |
| *TLR9 C>T rs187084* | | | | | | | | |
| Codominant |  |  |  |  |  |  |  |  |
| CC | 58 (71%) | 54 (77%) |  |  |  |  |  |  |
| CT | 24 (29%) | 15 (22%) | 0.67 (0.32 – 1.41) | 0.265 | 213.1 | 0.62 (0.28 – 1.37) | 0.184 | 206.1 |
| TT | 0 (0%) | 1 (1%) | - |  |  | - |  |  |
| Dominant |  |  |  |  |  |  |  |  |
| CC | 58 (71%) | 54 (77%) | 0.72 (0.34 – 1.49) | 0.369 | 213.0 | 0.69 (0.32 – 1.48) | 0.335 | 206.6 |
| CT-TT | 24 (29%) | 16 (23%) |  |  |  |  |  |  |
| Recessive |  |  |  |  |  |  |  |  |
| CC-CT | 82 (100%) | 69 (99%) |  |  |  |  |  |  |
| TT | 0 (0%) | 1 (1%) | - | - | - | - | - | - |
| Overdominant |  |  |  |  |  |  |  |  |
| CC-TT | 58 (71%) | 55 (79%) | 0.66 (0.31 – 1.39) | 0.267 | 212.5 | 0.62 (0.28 – 1.35) | 0.221 | 206.0 |
| CT | 24 (29%) | 15 (21%) |  |  |  |  |  |  |
| log-Additive  0,1,2 | 82 (54%) | 70 (46%) | 0.79 (0.39 – 1.59) | 0.265 | 213.3 | 0.77 (0.37 – 1.62) | 0.490 | 207.0 |
| *TLR9 C>T rs187084* | | | | | | | | |
| Codominant |  |  |  |  |  |  |  |  |
| TT | 25 (31%) | 25 (36%) |  |  |  |  |  |  |
| CT | 33 (40%) | 24 (33%) | 0.73 (0.34 -1.56) | 0.712 | 215.1 | 0.67 (0.30 – 1.48) | 0.586 | 208.5 |
| CC | 24 (29%) | 21 (30%) | 0.88 (0.39 – 1.96) |  |  | 0.80 (0.39 – 2.08) |  |  |
| Dominant |  |  |  |  |  |  |  |  |
| TT | 25 (30%) | 25 (36%) | 0.79 (0.52 – 2.08) | 0.494 | 213.3 | 0.76 (0.38 – 1.54) | 0.450 | 207.0 |
| CT-CC | 57 (70%) | 45 (64%) |  |  |  |  |  |  |
| Recessive |  |  |  |  |  |  |  |  |
| TT-CT | 58 (71%) | 49 (70%) |  |  |  |  |  |  |
| CC | 24 (29%) | 21 (30%) | 1.04 (0.52 – 2.08) | 0.921 | 213.8 | 1.11 (0.54 – 2.29) | 0.776 | 207.4 |
| Overdominant |  |  |  |  |  |  |  |  |
| TT-CC | 49 (60%) | 46 (66%) | 0.77 (0.40 – 1.50) | 0.448 | 213.2 | 0.70 (0.35 – 1.40) | 0.316 | 206.5 |
| CT | 33 (40%) | 24 (34%) |  |  |  |  |  |  |
| log-Additive  0,1,2 | 82 (54%) | 70 (46%) | 0.93 (0.62 – 1.39) | 0.726 | 213.6 | 0.94 (0.62 – 1.43) | 0.775 | 207.4 |

^a^Adjusted for sex and age; ^b^OR: Odds Ratio; ^c^p value: < 0.05; ^d^95% confidence interval; ^e^AIC: Akaike information criterion value.
