# Supplementary material for: Association of Toll-like receptors polymorphisms with the risk of acute lymphoblastic leukemia in the Brazilian Amazon
Source: Sci Rep. 2022 Sep 7;12:15159. doi: 10.1038/s41598-022-19130-7 (PMC9452670; doi:10.1038/s41598-022-19130-7)
Supplement: Supplementary file 4 — Supplementary Information 4. [file 41598_2022_19130_MOESM4_ESM.docx]

**Supplementary Table 3.** Analysis of the association of single nucleotide polymorphisms (SNPs) in study with infectious comorbidities in acute lymphoblastic leukemia patients.

|  | Infectious comorbidities | | | | | | | |
| --- | --- | --- | --- | --- | --- | --- | --- | --- |
| **Genetic**  **models** | **No**  **n=88 (%)** | **Yes**  **n=64 (%)** | **OR (95% CI)** | ***p* value** | **AIC** | **OR (95% CI)**  **adj** | ***p* value**  **adj** | **AIC** |
| ***TLR1 T>G rs5743618*** | | | | | | | | |
| Codominant |  |  |  |  |  |  |  |  |
| TT | 54 (61%) | 32 (50%) |  |  |  |  |  |  |
| TG | 27 (31%) | 28 (44%) | 1.75 (0.88 – 3.48) | 0.255 | 210.2 | 1.79 (0.90 – 3.58) | *0.234* | 213.8 |
| GG | 7 (8%) | 4 (6%) | 0.96 (0.26 – 3.55) |  |  | 0.98 (0.27 – 3.61) |  |  |
| Dominant |  |  |  |  |  |  |  |  |
| TT | 54 (61%) | 32 (50%) | 1.59 (0.83 – 3.05) | 0.162 | 209.0 | 1.62 (0.84 – 3.13) | *0.148* | 212.6 |
| TG-GG | 44 (49%) | 32 (50%) |  |  |  |  |  |  |
| Recessive |  |  |  |  |  |  |  |  |
| TT-TG | 81 (92%) | 60 (94%) |  |  |  |  |  |  |
| GG | 7 (8%) | 4 (6%) | 0.77 (0.22 – 2.76) | 0.686 | 240.7 | 0.78 (0.22 – 2.77) | *0.692* | 214.6 |
| Overdominant |  |  |  |  |  |  |  |  |
| TT-GG | 61 (69%) | 36 (56%) | 1.76 (0.90 – 3.43) | 0.098 | 208.2 | 1.79 (0.91 – 3.53) | *0.088* | 211.8 |
| TG | 27 (31%) | 28 (44%) |  |  |  |  |  |  |
| Log-Additive  0,1,2 | 88 (58%) | 64 (42%) | 1.28 (0.77 – 2.13) | 0.350 | 210.0 | 1.29 (0.77 – 2.16) | *0.329* | 213.8 |
| ***CD14 C>T*** ***rs2569191*** | | | | | | | | |
| Codominant |  | | |  |  |  |  |  |
| CC | 19 (22%) | 20 (31%) |  |  |  |  |  |  |
| CT | 53 (60%) | 32 (50%) | 0.57 (0.27 – 1.23) | 0.361 | 210.9 | 0.56 (0.26 – 1.22) | 0.335 | 214.5 |
| TT | 16 (18%) | 12 (19%) | 0.71 (0.27 – 1.89) |  |  | 0.71 (0.26 – 1.91) |  |  |
| Dominant |  |  |  |  |  |  |  |  |
| CC | 19 (22%) | 20 (31%) |  |  |  |  |  |  |
| CT-TT | 69 (78%) | 44 (69%) | 0.61 (0.29 – 1.26) | 0.180 | 209.1 | 0.59 (0.28 – 1.25) | 0.168 | 212.8 |
| Recessive |  |  |  |  |  |  |  |  |
| CC-CT | 72 (82%) | 52 (81%) |  |  |  |  |  |  |
| CC | 16 (18%) | 12 (19%) | 1.04 (0.45 – 2.38) | 0.928 | 210.9 | 1.06 (0.46 – 2.44) | 0.894 | 214.7 |
| Overdominant |  |  |  |  |  |  |  |  |
| CC-TT | 35 (40%) | 32 (50%) |  |  |  |  |  |  |
| CT | 53 (60%) | 32 (50%) | 0.66 (0.34 – 1.26) | 0.210 | 209.3 | 0.64 (0.33 – 1.24) | 0.188 | 213.0 |
| Log-Additive  0,1,2 | 88 (58%) | 64 (42%) | 0.81 (0.50 – 1.32) | 0.401 | 210.2 | 0.81 (0.49 – 1.33) | 0.403 | 214.0 |
| ***TLR4 A>G rs4986790*** | | | | | | | | |
| Codominant |  |  |  |  |  |  |  |  |
| AA | 84 (95%) | 59 (92%) |  |  |  |  |  |  |
| AG | 3 (3%) | 8 (8%) | 2.37 (0.55 – 10.32) | 0.362 | 210.4 | 2.37 (0.54 – 10.31) | 0.273 | 214.1 |
| GG | 1 (2%) | - |  |  |  |  |  |  |
| Dominant |  |  |  |  |  |  |  |  |
| AA | 84 (95%) | 59 (92%) | 1.78 (0.46 – 6.91) | 0.403 | 210.2 | 1.75 (0.45 – 6.83) | 0.415 | 214.1 |
| AG-GG | 4 (5%) | 5 (8%) |  |  |  |  |  |  |
| Recessive |  |  |  |  |  |  |  |  |
| AA-AG | 87 (99%) | 64 (100%) |  |  |  |  |  |  |
| AA | 1 (1%) | - | - | - | - | - | - | - |
| Overdominant |  |  |  |  |  |  |  |  |
| AA-GG | 85 (96%) | 59 (92%) | 2.40 (0.55 – 10.44) | 0.233 | 209.5 | 2.40 (0.55 – 10.44) | 0.234 | 213.3 |
| AG | 3 (4%) | 5 (8%) |  |  |  |  |  |  |
| Log-Additive  0,1,2 | 88 (58%) | 64 (42%) | 1.32 (0.41 – 4.26) | 0.362 | 210.7 | 1.30 (0.40 – 4.20) | 0.663 | 214.5 |
| ***TLR4 C>T rs4986791*** | | | | | | | | |
| Codominant |  |  |  |  |  |  |  |  |
| CC | 81 (92%) | 63 (98%) |  |  |  |  |  |  |
| CT | 6 (7%) | 1 (2%) | 0.21 (0.03 – 1.83) | 0.239 | 209.1 | 0.21 (0.02 – 1.83) | 0.142 | 212.8 |
| TT | 1 (1%) | - |  |  |  |  |  |  |
| Dominant |  |  |  |  |  |  |  |  |
| CC | 81 (92%) | 63 (98%) | 0.18 (0.02 – 1.53) | 0.060 | 207.4 | 0.18 (0.02 – 1.50) | *0.058* | 211.1 |
| CT-TT | 7 (8%) | 1 (2%) |  |  |  |  |  |  |
| Recessive |  |  |  |  |  |  |  |  |
| CC -CT | 87 (99%) | 64 (100%) | - | - | - | - | - | - |
| TT | 1 (1%) | - |  |  |  |  |  |  |
| Overdominant |  |  |  |  |  |  |  |  |
| CC-TT | 82 (93%) | 63 (98%) | 0.22 (0.03 – 1.85) | 0.103 | 208.3 | 0.21 (0.02- 1.85) | 0.103 | 212.1 |
| CT | 6 (7%) | 1 (2%) |  |  |  |  |  |  |
| Log-Additive  0,1,2 | 88 (58%) | 64 (42%) | 0.20 (0.03 – 1.56) | 0.239 | 207.2 | 0.19 (0.02 – 1.53) | 0.504 | 210.9 |
| ***TLR5 R>S rs5744105*** | | | | | | | | |
| Codominant |  |  |  |  |  |  |  |  |
| RR | 80 (91%) | 62 (97%) |  |  |  |  |  |  |
| RS | 8 (9%) | 2 (3%) | 0.32 (0.07 – 1.57) | 0.126 | 208.6 | 0.33 (0.07 – 1.62) | 0.137 | 212.5 |
| log-Addtive  0,1,2 | 88 (58%) | 64 (42%) | 0.32 (0.07 – 1.57) |  | 208.6 | 0.33 (0.07 – 1.62) |  | 212.5 |
| ***TLR6 C>T rs5743810*** | | | | | | | | |
| Codominant |  |  |  |  |  |  |  |  |
| TT | 63 (71%) | 44 (69%) |  |  |  |  |  |  |
| CT | 20 (23%) | 11 (17%) | 0.79 (0.34 – 1.81) | 0.183 | 209.5 | 0.77 (0.33 – 1.80) | 0.164 | 213.1 |
| CC | 5 (5%) | 9 (14%) | 2.58 (0.81 – 8.21) |  |  | 2.67 (0.83 – 8.56) |  |  |
| Dominant |  |  |  |  |  |  |  |  |
| TT | 63 (72%) | 44 (69%) | 1.15 (0.57 – 2.31) | 0.705 | 210.8 | 1.15 (0.57 – 2.34) | 0.693 | 214.6 |
| CT-CC | 25 (28%) | 20 (31%) |  |  |  |  |  |  |
| Recessive |  |  |  |  |  |  |  |  |
| TT-CT | 83 (94%) | 55 (86%) |  |  |  |  |  |  |
| CC | 5 (6%) | 9 (14%) | 2.72 (0.86 – 8.54) | 0.079 | 207.8 | 2.81 (0.89 – 8.91) | 0.071 | 211.5 |
| Overdominant |  |  |  |  |  |  |  |  |
| TT-CC | 68 (77%) | 53 (83%) | 0.71 (0.31 – 1.60) | 0.399 | 210.2 | 0.70 (0.30 – 1.60) | 0.387 | 214.0 |
| CT | 20 (23%) | 11 (17%) |  |  |  |  |  |  |
| log-Additive  0,1,2 | 88 (58%) | 64 (42%) | 1.30 (0.79 – 2.13) | 0.294 | 209.8 | 1.31 (0.80 – 2.15) | 0.281 | 213.6 |
| ***TLR9 C>T rs187084*** | | | | | | | | |
| Codominant |  |  |  |  |  |  |  |  |
| CC | 65(74%) | 59 (71%) |  |  |  |  |  |  |
| CT | 23 (26%) | 23 (28%) | 0.96 (0.46 – 2.02) | 0.682 | 211.2 | 0.94 (0.44 – 1.99) | 0.409 | 214.9 |
| TT | 0 (0%) | 1 (1%) | - | - | - | - | - | - |
| Dominant |  |  |  |  |  |  |  |  |
| CC | 65 (74%) | 59 (71%) |  |  |  |  |  |  |
| CT-TT | 23 (26%) | 24 (29%) | 1.02 (0.49 – 2.12) | 0.953 | 210.9 | 1.01 (0.48 – 2.11) | 0.986 | 214.7 |
| Recessive |  |  |  |  |  |  |  |  |
| CC-CT | 88(100%) | 82 (98%) |  |  |  |  |  |  |
| TT | 0 (0%) | 1 (2%) | - | - | - | - | - | - |
| Overdominant |  |  |  |  |  |  |  |  |
| CC-TT | 65 (74%) | 48 (75%) | 0.94 (0.45 – 1.97) | 0.874 | 210.9 | 0.92 (0.44 – 1.96) | 0.838 | 214.7 |
| CT | 23 (26%) | 16(25%) |  |  |  |  |  |  |
| log-Additive  0,1,2 | 88 (58%) | 64 (42%) | 1.10 (0.55 – 2.21) | 0.686 | 210.8 | 1.09 (0.54 – 2.20) | 0.820 | 214.7 |
| ***TLR9 C>T rs5743836*** | | | | | | | | |
| Codominant |  |  |  |  |  |  |  |  |
| TT | 24 (27%) | 26 (40%) |  |  |  |  |  |  |
| CT | 36 (41%) | 21 (33%) | 0.54 (0.25 – 1.17) | 0.224 | 209.9 | 0.54 (0.25 – 1.18) | 0.233 | 213.8 |
| CC | 28(32%) | 17 (27%) | 0.56 (0.25 – 1.28) |  |  | 0.56 (0.25 – 1.28) |  |  |
| Dominant |  |  |  |  |  |  |  |  |
| TT | 24 (27%) | 26 (41%) | 0.55 (0.28 – 1.09) | 0.084 | 207.9 | 0.55 (0.28 -1.10) | 0.088 | 211.8 |
| CT-CC | 64 (73%) | 38 (59%) |  |  |  |  |  |  |
| Recessive |  |  |  |  |  |  |  |  |
| TT-CT | 60 (68%) | 47 (73%) |  |  |  |  |  |  |
| TT | 28 (32%) | 17 (27%) | 0.71 (0.36- 1.38) | 0.482 | 210.4 | 0.77 (0.38 – 1.59) | 0.484 | 214.2 |
| Overdominant |  |  |  |  |  |  |  |  |
| TT-CC | 52 (59%) | 43 (67%) |  |  |  |  |  |  |
| CT | 36 (41%) | 21 (33%) | 0.74 (0.49 – 1.12) | 0.307 | 209.9 | 0.71 (0.36 – 1.39) | 0.316 | 213.7 |
| log-Additive  0,1,2 | 88 (58%) | 64(42%) | 0.74 (0.49 – 1.12) | 0.150 | 208.8 | 0.74 (0,49 – 1.12) | 0.154 | 212.7 |

^a^Adjusted for sex and age; ^b^OR: Odds Ratio; ^c^p value: < 0.05; ^d^95% confidence interval; ^e^AIC: Akaike information criterion value.
