# Supplementary material for: Association of Toll-like receptors polymorphisms with the risk of acute lymphoblastic leukemia in the Brazilian Amazon
Source: Sci Rep. 2022 Sep 7;12:15159. doi: 10.1038/s41598-022-19130-7 (PMC9452670; doi:10.1038/s41598-022-19130-7)
Supplement: Supplementary file 5 — Supplementary Information 5. [file 41598_2022_19130_MOESM5_ESM.docx]

**Supplementary Table 5.** Association of the TLR and CD14 alleles with infectious comorbidities and death in patients with acute lymphoblastic leukemia.

|  | | Controls vs. ALL | | | | Infectious comorbidities | | | | Death | | | |
| --- | --- | --- | --- | --- | --- | --- | --- | --- | --- | --- | --- | --- | --- |
| SNV | **Allele** | **Controls**  **(n= 187)** | **ALL cases**  **(n=152)** | **OR**  **(IC 95%)** | ***p* value** | **No**  **(n= 88)** | **Yes**  **(n= 64)** | **OR**  **(IC 95%)** | ***p* value** | **No**  **(n=82)** | **Yes**  **(n=70)** | **OR**  **(IC 95%)** | ***p* value** |
| *TRL1 T>G*  *rs5743618* | T | 276 (71%) | 227 (75%) | 0.95 (0.67 – 1.35) | 0.795 | 135 (76%) | 92 (71%) | 1.28 (0.76 – 2.16) | 0.339 | 120 (73% | 107 (76%) | 0.84 (0.49 – 1.41) | 0.515 |
|  | G | 98 (29%) | 77 (25%) |  |  | 41 (24%) | 36 (29%) |  |  | 44 (27%) | 33 (24%) |  |  |
| *CD14 C>T*  *rs2569191* | C | 203 (54%) | 163 (53%) | 1.03 (0.76 – 1.40) | 0.804 | 91 (51%) | 72 (56%) | 0.83 (0.52 – 1.31) | 0.432 | 88 (53%) | 75 (53%) | 1.00 (0.63 – 1.57) | 0.987 |
|  | T | 169 (46%) | 141 (47%) |  |  | 85 (49%) | 56 (44%) |  |  | 76 (47%) | 65 (47%) |  |  |
| *TRL4 A>G*  *rs4986790* | A | 364 (97%) | 295 (97%) | 1.11 (0.44 – 2.76) | 0.822 | 168 (95%) | 127 (99%) | 0.16 (0.02 – 1.33) | 0.085 | 159 (96%) | 136 (97%) | 0.93 (0.24 – 3.55) | 1.000 |
|  | G | 10 (3%) | 8 (3%) |  |  | 8 (5%) | 1 (1%) |  |  | 4 (4%) | 4 (3%) |  |  |
| *TRL4 C>T*  *rs4986791* | C | 367 (98%) | 294 (96%) | 1.78 (0.67 – 4.74) | 0.240 | 168 (95%) | 127 (99%) | 2.17 (0.69 – 6.79) | 0.173 | 156 (95%) | 138 (98%) | 0.28 (0.05 – 1.35) | 0.114 |
|  | T | 7 (2%) | 3 (4%) |  |  | 7 (5%) | 1 (1%) |  |  | 8 (5%) | 2 (2%) |  |  |
| *TRL5 R>S*  *rs5744105* | R | 364 (97%) | 294 (96%) | 1.23 (0.50 – 3.01) | 0.637 | 168 (95%) | 126 (96%) | 0.33 (0.07 – 1.59) | 0.200 | 159 (96%) | 135 (96%) | 1.17 (0.33 – 4.15) | 1.000 |
|  | S | 10 (3%) | 10 (4%) |  |  | 8 (5%) | 2 (4%) |  |  | 5 (4%) | 5 (4%) |  |  |
| *TRL6 C>T*  *rs5743810* | C | 303 (81%) | 245 (80%) | 0.97 (0.66 – 1.42) | 0.889 | 146 (82%) | 99 (77%) | 1.42 (0.80 – 1.48) | 0.221 | 125 (76%) | 120 (85%) | 0.53 (0.29 – 0.96) | ***0.036*** |
|  | T | 71 (19%) | 59 (20%) |  |  | 30 (18%) | 19 (33%) |  |  | 39 (24%) | 20 (15%) |  |  |
| *TRL9 C>T*  *rs187084* | C | 308 (82%) | 41 (13%) | 29.93 (19.60 – 45.69) | 3.242 | 153 (86%) | 141 (82%) | 1.17 (0.64 – 2.17) | 0.596 | 140 (85%) | 123 (87%) | 0.80 (0.41 – 1.57) | 0.526 |
|  | T | 66 (18%) | 263 (86%) |  |  | 23 (14%) | 24 (18%) |  |  | 24 (15%) | 17 (15%) |  |  |
| *TRL9 C>T*  *rs5743836* | C | 224 (59%) | 157 (51%) | 1.39 (1.03 – 1.89) | ***0.031*** | 84 (47%) | 73 (57%) | 0.68 (0.43 – 1.08) | 0.109 | 83 (51%) | 74 (52%) | 0.91 (0.58 – 1.43) | 0.695 |
|  | T | 150 (41%) | 147 (49%) |  |  | 92 (53%) | 55 (43%) |  |  | 81 (49%) | 66 (48%) |  |  |

^a^OR: Odds Ratio; ^b^p value: < 0.05; ^c^95% confidence interval.
